# Bone Metastases of Endometrial Carcinoma Treated by Surgery: A Report on 13 Patients and a Review of the Medical Literature

**DOI:** 10.3390/ijerph19116823

**Published:** 2022-06-02

**Authors:** Jingyuan Wang, Yibo Dai, Tao Ji, Wei Guo, Zhiqi Wang, Jianliu Wang

**Affiliations:** 1 Department of Obstetrics and Gynecology, Peking University People’s Hospital, Beijing 100044, China; wjywangjingyuan@bjmu.edu.cn (J.W.); daiyibo@pku.edu.cn (Y.D.); wangjianliu@pkuph.edu.cn (J.W.); 2 Department of Orthopaedic Oncology, Peking University People’s Hospital, Beijing 100044, China; jitaomd@163.com (T.J.); bonetumor@163.com (W.G.)

**Keywords:** endometrial cancer, bone metastasis, surgery

## Abstract

Background: The aim of this study was to describe the clinicopathological features of endometrial cancer (EC) patients with bone metastases treated with surgery and to systematically review the literature. Methods: We performed a retrospective study to include patients with bone metastases of EC at Peking University People’s Hospital from 2000 to 2019. Clinicopathological features and survival outcomes were collected. Results: Among the 1662 patients with EC, 14 (0.84%) were identified with bone metastases, and all were treated surgically. Thirteen cases were analyzed. Four had bone metastases when diagnosed, and the remaining nine cases had bone metastases when first relapsed, with a median time to recurrence of 13 months (range, 5–144). The median age of the 13 patients was 58 years old (range, 45–76). Twelve were endometrioid carcinoma. The majority of sites of bone metastases were the pelvis, followed by the spine. The median overall survival (OS) was 57 months. We further combined the 13 patients with another 24 cases identified from literature research. There was no significant difference in clinicopathological characteristics between the patients with bone metastases when diagnosed and when they first relapsed. The median OS was numerically longer for patients with bone metastases when diagnosed than when they first relapsed (57 vs. 36 months, *p* = 0.084). Conclusions: Patients with bone metastases of EC might benefit from comprehensive treatment based on surgery, as symptoms can be palliated and survival can probably be extended.

## 1. Introduction

Endometrial cancer (EC) is one of the most common gynecological cancers in the world, and its incidence has increased remarkably in recent decades, with approximately 63,230 new cases in 2018 [1].

In general, EC recurrence mostly occurs within the pelvis, with a recurrence rate following initial treatment of around 11–13% [2]. EC is more likely to metastasize to the lymph nodes, liver and lungs [3]. In contrast, bone involvement is quite rare, and its prevalence is reported between 0% and 15% [4,5]. The mechanism of metastasis of EC is thought to be related to hematologic dissemination by the retrograde flow of tumor emboli or Batson’s paravertebral valveless venous plexus [6]. However, the exact mechanism is not fully clear.

Bone metastases cause severe suffering to patients because of severe pain, physical disability and pathologic skeletal-related events. Thus, early diagnosis and appropriate therapy are needed to enhance the quality of life (QOL) of patients with osseous dissemination. However, because of its rarity, the factors influencing the survival and optimal therapeutic regime of primary bone metastases in patients with EC are not known yet. Some support noninvasive measures, while others suggest surgery can prolong the cancer-specific survival of patients due to the resistance to chemotherapy or radiotherapy. It was established that pain, pathological fracture and spinal cord compression are all indications for surgery [7]. All cases in our study were diagnosed according to symptoms, and all these patients had pain at the site of the bones involved. Thus, all of them were treated surgically.

The main purpose of this study is to describe the clinicopathological features and treatment results of the primary bone metastases of EC treated by surgery.

## 2. Materials and Methods

### 2.1. Patients

This was a retrospective study including EC patients with osseous disseminations. Patients diagnosed with EC were identified from January 2000 to December 2019 in Peking University People’s Hospital. Afterwards, we particularly identified those patients who were diagnosed as primary bone metastases, including those who had bone metastases either at presentation with EC simultaneously or as the first site of recurrence (with or without concomitant extra-osseous lesions). Those with osseous dissemination that was subsequent locations of EC relapse were excluded. Patients with uterine sarcomas or carcinosarcomas were also excluded from this study. This study was approved by the Institutional Review Board of Peking University People’s Hospital (2020PHB331-01). All patients provided written informed consent.

### 2.2. Follow Up

In the first year after surgery, the patients were examined every one to two months, then every three to six months for the next three years, and yearly afterwards, including routine and pelvic examinations, smear examinations of the upper part of the vagina, abdominopelvic ultrasonography and evaluations of serous CA125. In the first two years, a chest X-ray was carried out every six months, and then every year for the next three years. If necessary, a thorough evaluation was applied to make use of computed tomography (CT), pelvic magnetic resonance imaging (MRI) or positron emission tomography-computed tomography (PET-CT) scan of the chest, abdomen and pelvis.

The primary functional outcome was defined as an improvement in specific pains, and a visual analog pain scale (VAS) was used to evaluate the degree of pain both before and after surgery. In order to minimize the impact of cancer recurrence on the primary outcome of pain improvement, we focused the analysis on immediate pre-operative assessment and that occurring in the initial three months after surgical intervention.

### 2.3. Data Collection

Clinicopathological characteristics and survival outcomes of patients with primary bone metastases of EC were obtained from medical records. Patients were staged according to the 2009 International Federation of Gynecology and Oncology (FIGO) criteria. Description of tumor data was abstracted from primitive pathological reports.

### 2.4. Literature Research

#### 2.4.1. Search Strategy

A systematic review was carried out adhering to the Preferred Reporting Items for Systematic Reviews and Meta-Analysis (PRISMA) guidelines. We searched PubMed and Embase databases from database inception to the present. We used the following search query: “((endometrial cancer OR endometrial malignancy OR endometrial neoplasm OR endometrial carcinoma) AND (bone OR skeletal) AND metasta*)”. The search terms were designed to identify all publications found in this field and chose those composed of clinical case series or case reports that described patients with primary bone metastases of EC treated by surgical approach. Only papers reported in English were included. Electronic searches were supplemented by reviewing reference lists of all articles identified in the primary search to obtain more complete data.

#### 2.4.2. Inclusion and Exclusion Criteria

Studies were included when the following criteria were met: cases on pathologically confirmed EC with primary bone metastasis; survival information was reported; cases treated with surgery; full study was available; studies obtained research ethics approval. Exclusion criteria were as follows: unavailable full text; review; republished literature; documents with incomplete original data and no corresponding data after contacting the author. Cases with osseous dissemination that was subsequent locations of EC relapse, or cases treated conservatively were also excluded.

#### 2.4.3. Data Extraction and Quality Assessment

After the removal of duplicates, two investigators screened titles and abstracts independently for initial study selection. After abstract screening, the two investigators reviewed the entire publication to determine whether it met the inclusion or exclusion criteria. Any disagreement was resolved between both investigators to reach a consensus, and a review agreement was finally achieved. The following data were obtained from original articles: (1) general information including author and year of publication; (2) clinicopathological information including age, histology type, pathological grade, stage, symptoms, interval to bone metastasis, localization, extraosseous metastatic localization, therapy and available outcome data. We assessed the quality of the included studies by adapting items from the Joanna Briggs Institute Reviews Manual (http://www.joannabriggs.org/sumari.html accessed on 10 May 2022) by two investigators independently.

### 2.5. Statistical Analysis

Clinicopathological characteristics were analyzed using descriptive statistics, and SPSS (version 24.0) was used for data management and statistical analysis. The median survival was estimated by the Kaplan–Meier method. The overall survival (OS) after bone metastases was defined as the time from diagnosis of osseous dissemination to death or last follow-up. Statistically significant difference was defined as *p* < 0.05.

## 3. Results

### 3.1. Patient Characteristics

Among the 1662 patients with EC diagnosed in Peking University People’s Hospital from 2000 to 2019, 14 (0.84%) were identified with bone metastasis, and all of them were treated surgically. One patient was lost of follow-up after surgery. Therefore, the clinicopathological and survival data of the remaining 13 patients were finally reviewed. The characteristics of patients are described in detail in Table 1.

Among the 13 patients, 4 patients had bone metastases identified simultaneously with the diagnosis of EC. The remaining 9 patients had bone metastases at the primary recurrence of EC, with the median time from diagnosis to bone metastasis of 13 months (range, 5–144 months). The median age was 58 years old (range, 45–76 years old). Except for one case of clear cell carcinoma, the remaining 12 cases were endometrioid carcinoma.

### 3.2. Characteristics of Bone Metastasis

All cases were diagnosed according to symptoms, and all these patients had pain at the site of the bones involved. The total locations of bone metastases were 23 in these 13 patients, and the maximum amount of osseous dissemination found in a single patient was three. The majority of sites of bone metastasis were pelvic bones (17/23 sites), followed by the spine (5/23 sites). In five patients, the distribution of bone metastases involved the axial skeleton. Six patients had other metastases, including the lungs, the skin and the abdomen, besides bone metastasis. Five patients had a single bone lesion, and eight had multiple bone metastases. Only one case was found to have a single bone site and no extra-osseous dissemination. All patients were treated with surgical resection with or without adjuvant therapy as the salvage treatment after osseous dissemination. Four cases were treated with chemotherapy monotherapy in two patients, chemotherapy plus radiotherapy in one patient and chemotherapy plus radiotherapy and hormone therapy in one patient. Six patients died of the disease at the date of data analysis. The median OS after diagnosis of bone metastases was 57 months.

The mean VAS pain scale score was 5.6 (SD 1.5) preoperatively compared with 2.3 (SD 2.1) three months after the operation. There was a significant improvement in pain three months after surgical intervention (*p* < 0.001). Pain relief was observed in 12 patients (92.3%).

### 3.3. Literature Review

**Figure 1:**
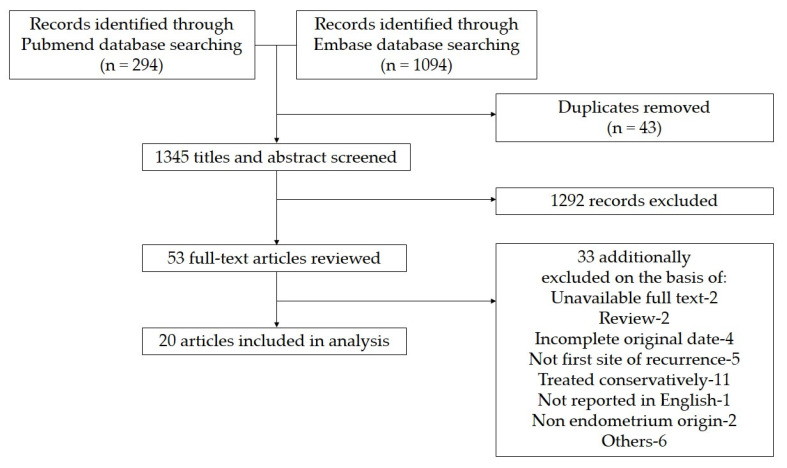
Flow diagram of the studies’ selection.

We further identified 20 studies [5,6,8,9,10,11,12,13,14,15,16,17,18,19,20,21,22,23,24,25] with a total of 24 cases of EC patients with bone metastases treated by surgery through literature research. The process of study recruitment is shown in Figure 1, and the detailed information on the included studies is displayed in Table 2. With the addition of our 13 cases, a sum of 37 cases was further analyzed. We then classified these cases into two groups: 24 cases with bone metastases when first relapsed and the other 13 cases with bone metastases when diagnosed as EC.

We then compared the clinicopathological features and the survival outcome between the two groups. The overall characteristics of the 37 patients are summarized in Table 3. In 24 cases, the median interval from the diagnosis of the EC to osseous dissemination was 18 months (range, 2 to 144 months). The predominant histopathological type was endometrioid carcinoma. Presenting symptoms were bone pain, swelling, throbbing, weakness, erythema, infection and lack of strength and sensation. Only 1 case was diagnosed as bone metastases without symptoms at the site involved. The most common affected location was the pelvic bones (23/59 sites (38.98%)). Bone recurrences were also seen in the vertebra, tarsus, talus, calcaneus, cranium, toe, sternum, femur, tibia, humerus, clavicle and ribs. At the diagnosis of bone metastasis, 11 (29.73%) patients had coexisting extra-osseous metastatic lesions, with the lungs (9/11) being the most frequent.

There was no significant difference in clinicopathological characteristics and survival outcomes between the two groups. The median OS after diagnosis of bone metastases for patients with bone metastases when first relapsed was 36 months, with the survival rate at 1 year, 2 years and 5 years being 79%, 59% and 31%, respectively, and the median OS for patients with bone metastases when diagnosed was 57 months, with the survival rate at 1 year, 2 years and 5 years being 92%, 92% and 46%, respectively. The median survival outcome of patients with bone metastasis, when diagnosed, was numerically longer than patients with bone metastasis when they first relapsed, while there was no significant difference (*p* = 0.084). In addition, the OS after bone metastasis was significantly longer in patients with endometrioid carcinoma than in those with type II carcinoma (57 vs. 4 months, *p* = 0.003). However, this may not be representative due to the small number of cases with type II carcinoma. Other clinicopathological features, including age, number of bone lesions, concomitant extra-osseous metastases, single bone involvement and no extra-osseous spread and metastases to the axial skeleton, were not associated with survival outcome.

## 4. Discussion

In the present study, we described the clinicopathological characteristics and survival outcomes of 13 EC patients with bone metastasis. We further combined the 13 patients with the 24 cases with bone metastasis identified from literature research. We found that the median OS after bone metastases was significantly associated with histology.

The occurrence of osseous dissemination secondary to EC is rarely seen, and the incidence is unknown. Although autopsy data shows an incidence up to 25%–27% [26], the clinical frequency is reported to be only 0.4%–1.8% [20,22,27]. Uccella et al. [22] also pointed out that the incidence of osseous dissemination in EC was less than 1%, according to the literature review. In contrast, in Takeshita’s cohort study [28], the incidence rate was up to 3.1%. The difference in sources of patients admitted to research centers might contribute to this distinction. In our series, the total incidence of bone metastases was 0.84% and 0.24% in patients with bone metastases at presentation with EC.

Although with no significant difference statistically, the survival outcome was better for the patients with osseous dissemination as a presenting feature of EC than as the first site of recurrence (57 vs. 36 months) in our study. The same result was reached in Vizzielli et al. [21] and Uccella et al.’s [22] research (28 vs. 21 months, and 20 vs. 10.5 months, respectively). In addition, the limited sample size of this study may not be sufficient enough to obtain significant results, and further research is needed to determine the clinical significance of patterns of osseous dissemination.

At the time of diagnosis, most patients with bone metastasis had pain [25]. In our study, all patients presented with symptoms of pain, and in our literature review, bone dissemination was discovered incidentally only in one case. The sacrum metastasis of the patient was found by a conventional follow-up computed tomography scan 37 months after the operation, and confirmation was made by computed tomography-guided biopsy. Given the low incidence of bone metastasis, there is no routine assessment of bone dissemination in the surveillance of EC. Despite this, there is a need to have a suspicion for metastasis in EC patients presenting bone tenderness and in patients with no history of cancer but with osteodynia responding poorly to conservative management, as EC may be an underlying cause [4]. The initial diagnosis may be difficult since the symptom of bone pain is more common in benign diseases such as trauma, soft tissue inflammation, arthritis and osteomyelitis [29]. While there is no standard approach to diagnose bone disease, plain radiograph, bone scan, MRI, positron emission tomography (PET), aspiration cytology and bone biopsy may help in the diagnosis. It is reported that technetium diphosphonate bone scans can be positive 18 months earlier before a lesion is detected on plain X-ray [30]. CT or MRI can be more helpful than bone scans since these methods may be useful to help diagnose other metastatic locations, which could change the therapeutic scheme [20]. PET/CT scans are used to determine malignancies of bone lesions, similar to the CT scans for other malignant tumors, including breast cancer [31]. Therefore, we propose that clinical and radiologic assessments should be carried out for patients with suspected lesions to rule out bone metastasis.

Most of the tumors that metastasize to the bones are endometrioid [6], instead of those histologic subtypes supposed to be more aggressive, such as papillary serous or clear cell carcinomas. In our study, the predominant histopathological type was endometrioid (86%, 32/37), 57% (21/37) of the tumors were moderately or poorly differentiated and 41% (15/37) were in FIGO stage IV. Some studies postulate that type II EC is associated with a worse prognosis [22,27] and is a predictor of hematogenous dissemination in EC [32]. Our study reached a similar conclusion that endometrioid carcinoma was associated with longer survival despite the small number of cases with type II carcinoma. It is suggested that EC with advanced stage and poorly differentiated grade is more likely to metastasize to bone [20]. Though rare, reports exist concerning bone recurrence in early-stage and well-differentiated EC [5]. In fact, different types of EC have specific histopathological and molecular characteristics. Traditional pathological analysis remains an important tool for risk stratification but brings the problem of undertreatment or overtreatment. The Cancer Genome Atlas (TCGA) Research Network identified four molecular subgroups of endometrial carcinoma: copy-number-low/p53-wild-type (p53wt), POLE-mutated/ultramutated (POLEmt), microsatellite-instability/hypermutated (MSI) and copy-number-high/p53-mutated (p53mt) in 2013 [33]. The high costs and complex technology required for sequencing techniques limited the application of the TCGA classification in clinical practice [34,35,36]. Therefore, the Proactive Molecular Risk Classifier for Endometrial Cancer (ProMisE) was developed to identify distinct molecular subgroups based on a combination of mutation analysis and immunohistochemistry [37]. Four molecular subtypes were identified, and this surrogate approach has the potential to improve the risk stratification in endometrial carcinoma. The subgroup of DNA polymerase epsilon (POLE), with mutations in the exonuclease domain of Polymerase-ε, shows an excellent prognosis, while the subgroup of p53 is abnormal (p53abn), with aberrant p53 immunohistochemical staining, demonstrates the worst prognosis. ECs with MMR-D, showing loss of one or more mismatch repair protein(s), or p53 wild-type (p53wt) have an intermediate prognosis [36]. In addition, traditional histological results of EC have significant value for the risk stratification independently of the molecular characterization, with non-endometrioid carcinoma having poor outcomes in each TCGA molecular subgroup. An integrated prognostic risk stratification combining traditional histological factors and molecular results was proposed in the European Society of Gynecological Oncology, European Society for Radiotherapy and Oncology and European Society of Pathology (ESGO/ESTRO/ESP) guidelines to assess the prognosis of EC [38,39]. Specifically, the prognosis of the MSI subgroup was the worst among non-endometrioid carcinomas, followed by non-endometrioid p53mt carcinomas. However, non-endometrioid carcinomas in the POLEmt subgroup showed a favorable prognosis instead. Considering only endometrioid histotypes, the survival outcome was different amongst the TCGA subgroup. ECs of the p53mt subgroup showed the worst prognosis, while the prognosis was the best in the POLEmt subgroup; endometrioid MSI carcinomas showed a significant overlap with the p53wt group [39]. These findings indicate that the histotype remains to have a strong impact on the prognosis in EC, and integration with molecular characterization for the risk stratification is worthy of support to optimize treatment [40,41]. In addition, there are other pathological features such as grade, myometrial invasion and lymphovascular space invasion (LVSI), which are proposed as independent prognostic factors [42,43,44,45,46]. Thus, a more tailored therapeutic regimen for EC patients can be achieved based on a combined risk assessment of these molecular and pathological prognostic factors. Stage I p53mt ECs are classified as intermediate risk or high risk according to the myometrial invasion status. For p53mt ECs without myometrial invasion, adjuvant treatment may be considered. However, for p53mt carcinomas with deep myometrial invasion, adjuvant treatment is strongly recommended. Instead, myometrial invasion has little effect on POLE-mt ECs prognostically [38]. Stage IA MMR-D ECs with negative or focal LVSI are classified as low risk following regular follow-up. On the other hand, stage IB MMR-D ECs will be upgraded from intermediate to high–intermediate with the presence of LVSI, and adjuvant treatment is recommended in these cases [47]. However, no effect of LVSI on the risk stratification were identified in POLE-mt and p53-mt ECs [38]. Moreover, further studies are needed to predict the risk of molecular factors to developing bone metastasis.

In some literature, the axial skeleton was the predominant metastatic site [20,22,27], while some researchers insisted that most bone metastases of EC affected the appendicular skeleton [21,48]. Similar to Gunsu Kimyon’s report [24], the involvement of the axial (46%) and appendicular (54%) skeleton was almost equally observed in our study. It showed that 70% of cases had single localization bone recurrence and most patients had isolated bone metastases without extra-osseous spread [6,23]. In the literature review, the most frequent sites of involvement were the vertebrate bones [48], with the pelvic bones being the second most common [21,22]. In our series, most patients had metastases in a single bone site (54%), and the pelvic bones (39%) were the most commonly affected bones. At the same time of diagnosis of osseous dissemination, 11 (30%) patients had coexisting extra-osseous metastatic lesions, with the lungs being the most frequent. In addition, Ucella et al. reported that patients with a single bone recurrence and the absence of extra-osseous spread have a better prognosis [22], and in some literature, the outcome appeared to be more favorable in those patients with isolated bone metastases [4,20]. However, we did not find a statistically significant relationship between these characteristics of bone metastases above and survival outcomes.

At present, the optimal therapeutic strategy has not been established yet for the patients with EC who developed bone metastases because of the few descriptions available in the literature due to their rarity and the various osseous sites being involved. Therapeutic schedules include surgical resection, directed radiotherapy, systemic chemotherapy and hormonal therapy if hormone receptor-positive. In accordance with literature from other solid tumors such as breast and prostate, another option as the treatment modality is a bisphosphonate (zoledronic acid) [49]. The selection of regimen differs according to previous therapy, the number and location of bones involved, concomitant extra-osseous dissemination and patient’s performance status [27].

The impact of surgery management on survival outcomes is not clear. Some researchers would argue that the tumor in the bone could just be a manifestation of a disseminated process and that it would be only a matter of time before other subclinical distant metastases became evident [50]. At the same time, the literature on the treatment of pulmonary metastases indicated that surgeons and oncologists treated some clinical criteria as a minimal standard of operability. These guidelines involve the feasibility of the complete removal of the primary lesion and all pulmonary metastatic tumors and the absence of verifiable extrathoracic metastases. In these conditions, resection of the lung metastases seems reasonable [51]. Imitating management, surgical resection of the bone metastases of EC may be justified. Thus, all patients were treated with wide resection and reconstruction to relieve symptoms palliatively, with or without adjuvant therapy in our study. The benefits of surgical treatment are well established, and the main objectives are the decompression of the spinal cord, better pain control, reconstruction and improvement in function and quality of life. Different reconstruction techniques are available, and the choice depends on the patient’s prognosis, size of the bone defect and the response of the tumor to adjuvant treatment.

When metastatic lesions involve the spine, the surgical resection and reconstruction of the vertebra should be undertaken following the division of Weinsteins. For patients undergoing vertebral body resection and subtotal resection, fixation through the anterior approach alone can support enough strength to reconstruct. For patients with total vertebrectomy, the stability of the spine is maintained through the anterior approach, combined with the posterior approach. If the tumor is confined to the vertebral body, accurate intraoperative localization and full exposure to the lesion is required. In the case of osteogenic metastases, a burr can be used to remove the lesions; for osteolytic metastases, curettes and suction devices are often used to remove the lesions. When removing the lesion, there should be no residual tumor until the posterior longitudinal ligament so as to relieve the compression on the dural sac. If there is nerve root compression, tumor resection and decompression around the nerve root should also be performed, and the resection of the tumor around the nerve root should also be performed to decompress the nerve root. According to the origin of the tumor and the degree of malignancy of the tumor, a bone graft or intramedullary needle bone cement is selected to fill the bone defect, and the corresponding anterior fixation is performed. If the appendix is also resected combined with the vertebral body, posterior Lugue rods or pedicle screws can also be used to enhance fixation in addition to anterior reconstruction. When vertebral body bone grafting or bone cement filling is used to reconstruct bone defects, care should be taken to avoid the compression of the posterior dural sac and the injury of important anterior vascular organs.

When metastatic lesions involve the pelvis, the surgery consists of the wide resection of en bloc removal of the bone lesion with an envelope of normal tissue and reconstruction with methy lacrylate alone or combined with Steinmann pins and a screw-rod system. The most common is the treatment of metastases around the acetabulum. Due to the short survival time and poor prognosis of patients, the treatment should be palliative rather than radical. Therefore, after the resection of metastatic acetabular tumors, simple and rapid methods are often used for pelvic and joint reconstruction. To achieve this goal, the best option is to use cement-based total hip replacement, which is often performed by curettage rather than mass excision. When total hip arthroplasty is used for acetabular metastases surgery, the acetabular part is usually fixed with bone cement. If the bone destruction is small and the displacement of the femoral head is not obvious, the application of ordinary bone cement acetabulum can achieve good results. The prosthesis of the femoral part can use conventional cemented total hip replacement stems.

In addition to EC, bone metastasis also occurs in other gynecological malignancies. Although the incidence of ovarian cancer is not as high as that of other cancers, such as EC, it is the most lethal of the female reproductive tract malignancies in the United States [52]. According to literature, the incidence of bone metastasis in ovarian cancer is less than 1%, with a median survival of fewer than eight months [53]. Moreover, the incidence is related to tumor stage, and bone metastasis is more common in advanced tumor [54]. However, the incidence is up to 10% based on the postmortem evaluation report [55]. In J Sehouli et al.’s study, the survival outcome was closely associated with the interval from the diagnosis of ovarian cancer to the occurrence of bone metastasis. When the interval was less than 12 months, the prognosis was poor (*p* < 0.01) [56]. The fact that patients would benefit from radiation or orthopedic surgery and the therapeutic effect of oral bisposphonates was affirmative. However, responsiveness to chemotherapy was not good. Bone metastasis occurred in 16% of patients with cervical cancer, with the lumbar and thoracic spine being the most frequently affected [57]. The prognosis of patients who received adjuvant chemotherapy or radiotherapy was better than that of the patients who received palliative care only. As for treatment, the combination of chemotherapy and radiotherapy was the most effective and radiotherapy can relieve pain effectively [58]. However, bone metastasis often occurs in the vicinity of previously irradiated areas; therefore, radiation therapy should be adhered to the indications strictly [59]. When gynecological malignancies metastasize to bone, the main purpose of treatment is to relieve pain and improve QOL. If indications have been met, palliative surgery can be selected, and adjuvant therapy can be undertaken postoperatively.

It is suggested that radiotherapy works on most occasions and may be curative [4,29]. Radiotherapy failed to improve survival due to the unresponsiveness of the aggressive tumors to the nonsurgical treatment [60]. However, the application of radiotherapy is still recommended to control local destruction and prevent fracture to the bone metastasis lesions [61]. In patients with single-site bone metastasis, local radiotherapy is curative indeed, while chemotherapy will be applied first in patients with multiple locations. Therefore, a combination of radiotherapy and chemotherapy is recommended, which may be useful in controlling tumor volume [62]. After that, zoledronic acid is chosen to relieve bony pain, although it does not inhibit tumor growth [63]. With regard to the treatment in our series, most of the cases operated on were treated with adjuvant therapy, while the other few did not.

In a review of the literature, a total of 37 cases who developed bone metastases of EC were treated by surgical approach with or without the adjuvant therapy, and the median survival was 57 months (range, 31–83 months). In contrast, according to previous literature reports, the median survival after the detection of osseous dissemination ranged from 10 months to 26 months [20,21,22]. Some experts reported that pain, pathological fracture and spinal cord compression were all indications for surgery [7]. When a cure is not achievable, palliation of symptoms should be a priority and pain control, a better performance grade and an improvement in QOL after operation of bone metastases were observed indeed [64]. In our study, the VAS was a significant index to assess the patient’s pain, and pain relief could be observed in 92.3% of patients according to VAS.

Moreover, the aggressive operation may prolong the median survival by keeping a good QOL. Similarly, the median survival of the 19 patients with primary bone metastases of EC treated at Mayo Clinic was 12 months, and only 1 of them received complete surgical excision and survived 41 months after the operation [22]. Currently, the issue of the best treatment plan remains controversial, but the main objective of treatment should be to palliate or even eliminate pain and extend survival. Taking the QOL into account, our case series may propose a probable therapeutic scheme of choice that the comprehensive treatment based on surgery be added to standard procedure for patients with bone dissemination of EC.

Our study was retrospective in nature, with the main limitations of the small study group and the heterogeneity of patients. Therefore, we acknowledge that it was not possible to draw any conclusions, but our results can be compared with various other published case reports in terms of clinicopathologic characteristics, treatment and survival outcome.

## 5. Conclusions

Patients with primary bone metastases of EC might benefit from comprehensive treatment based on surgery, as symptoms can be palliated and survival can probably be extended.

## Figures and Tables

**Table 1:** Characteristics of 13 patients with primary bone metastases of EC treated by surgery at Peking University People’s Hospital.

Pt. No.	Age (Years)	Histology, Grade, Stage	Symptoms at Presentation	CA-125 (u/mL)	Interval to Bone Met (Months)	No. of Bone Met	Side	Localization	Extraosseous Met	Palliative Surgical Resection	Adjuvant Therapy after Surgery	Status	Survival after Bone Met (Months)	VAS (Pre/Post-Operative)
1	45	ADK,G2,IV	pain	84.62	9	1	Median	T12	Skin	Reconstruction	/	Dead	1	7/3
2	54	ADK,NA,NA	Pain	NA	38	1	Median	L2	No	Reconstruction	/	Dead	20	4/1
3	58	Clear cell,G3,II	Pain	NA	48	2	L	Acetabulum, L1	No	Reconstruction	/	Dead	4	8/3
4	76	ADK,NA,III	Pain	242.6	10	1	Median	L3	Lung	Reconstruction	RT	Dead	4	6/4
5	55	ADK,NA,NA	Pain	56.12	13	1	R	Tibia	Lung	Reconstruction	/	Live	47	6/2
6	65	ADK,NA,NA	Pain	22.06	144	2	Bilateral	Ilium	No	Reconstruction	/	Dead	16	4/1
7	74	ADK,G2,I	Pain	7.79	13	3	R	Sacrum, ilium, L5	No	Reconstruction	/	Live	53	4/0
8	54	ADK,G2,IV	Pain	14.31	At dx	2	L	Pubis, ischium	No	Reconstruction	RT + CHT + HT	Dead	57	6/8
9	56	ADK,G3,IV	Pain	64.74	At dx	2	R	Pubis, ischium	No	Reconstruction	RT + CHT	Live	14	5/1
10	59	ADK,G3,IV	Pain	39.88	At dx	2	L	Pubis, ischium	No	Reconstruction	CHT	Live	12	3/1
11	63	ADK,G1,I	Pain	NA	5	3	R	Acetabulum, pubis, ischium	Lung	Reconstruction	/	Live	35	7/3
12	45	ADK,G3,IV	Pain	374.50	At dx	1	R	Pubis	Abdomen	Reconstruction	CHT	Live	18	7/2
13	72	ADK,NA,NA	pain	204.50	36	2	R	Acetabulum, pubis	Lung	Reconstruction	/	Live	4	6/1

Abbreviations: ADK, adenocarcinoma; CHT, chemotherapy; dx, diagnosis; HT, hormone therapy; L, left; met, metastases; NA, not available; Pt., pat1ient; R, right RT, radiotherapy; VAS, visual analog pain scale.

**Table 2:** Characteristics of 24 patients with primary bone metastases of EC treated by surgery reported in the literature.

Author	Pt. No.	Age (Years)	Histology, Grade, Stage	Symptoms at Presentation	Interval to Bone Met (Months)	Localization	Extraosseous Met	Therapy	Status	Survival after Bone Met (Months)
Ravault et al. [8]	1	61	NA,NA,NA	Pain	36	R tarsus	No	Surgery, RT	Live	7
Petru et al. [9]	2	61	ADK,G1,IV	Pain, swelling	At dx	L tarsus	No	Surgery, CHT, HT	Live	10
Clarke and Smith [10]	3	55	ADK,NA,NA	Pain, swelling	18	R talus, calcaneus	Lung	Surgery, RT	Dead	36
Mustafa et al. [11]	4	45	ADK,G2,I	Infection	36	Cranium	Lung, pelvic sidewall	Surgery, HT	Dead	6
Neto at al. [12]	5	39	ADK,G2,IV	Pain, tumble	At dx	R ischium	No	Surgery, RT	Live	36
Arnold et al. [13]	6	63	ADK,G1,IV	Pain, leg weakness	At dx	T12	No	Surgery, RT, HT	Live	60
Ali et al. [14]	7	77	ADK,G3,I	Throbbing, swelling	24	L 4th toe, distal phalanx	Lung	Surgery, HT	Live	16
Haraguchi et al. [15]	8	87	NA,NA,NA	Pain	108	Sternum	No	Surgery	Live	60
Uharcek et al. [16]	9	67	ADK,G1,IV	Pain, erythema, swelling	At dx	R foot	No	Surgery, CHT, HT	Live	20
Albareda et al. [5]	10	62	ADK,G1,I	None	37	Sacrum	No	Surgery, HT	Live	26
Qin et al. [17]	11	48	ADK,G3,II	Pain	22	R and L femur	No	Surgery, CHT, HT, RT	Live	42
Pakos et al. [18]	12	62	ADK,G3,II	Pain	7	R tibia	No	Surgery	Live	27
Chan et al. [19]	13	62	NA,NA,NA	Pain	3	Sternum	NA	Surgery	Dead	18
Kehoe et al. [20]	14	58	ADK,G3,I	Pain	10	L4, L5	No	Surgery, RT, CHT	Live	199
Kehoe et al. [20]	15	60	Clear cell,G3,NA	Pain	12	Humerus, clavicle	No	Surgery, RT, CHT	Dead	13
Kehoe et al. [20]	16	55	ADK,G3,III	Pain	9	Rib	No	Surgery, RT	Dead	26
Kehoe et al. [20]	17	55	ADK,G3,IV	Pain	At dx	Ischium, acetabulum, femur	No	Surgery, RT	Dead	10
Jiang et al. [6]	18	51	ADK,G2,IV	Pain, swelling	At dx	L tibia, calcaneus, tarsus	Lung	Surgery, CHT, HT	Live	56
Vizzielli et al. [21]	19	62	ADK,G1,IV	Pain	At dx	Thigh, acetabulum, ischiopubic bone	Lung	Surgery, CHT	Live	30
Uccella et al. [22]	20	65	ADK,G2,IV	Pain	19	R sternum	No	Surgery, HT	Dead	60
Uccella et al. [22]	21	65	ADK,G2,NA	Lack of strength and sensation	18	T5	No	Surgery, RT, HT	Dead	9
Myriokefalitaki et al. [23]	22	57	ADK,G2,IV	Pain	At dx	R femur	No	Surgery, RT	Live	53
Kimyon et al. [24]	23	62	ADK,G2,I	Pain	2	Tibia, femur	No	Surgery, RT, CHT	Live	22
Makris et al. [25]	24	68	ADK,G1,IV	Pain	At dx	R tibia	No	Surgery, RT, CHT	Live	6

Abbreviations: ADK, adenocarcinoma; CHT, chemotherapy; dx, diagnosis; HT, hormone therapy; L, left; met, metastases; NA, not available; Pt., patient; R, right; and RT, radiotherapy.

**Table 3:** Overall characteristics of 37 patients with primary bone metastases of EC treated by surgery.

Characteristics	Bone Metastases at Primary Recurrence of EC (n = 24)	Bone Metastases at the Diagnosis of EC (n = 13)	*p*-Value
Age, median (range), years	62 (45, 87)	57 (39, 68)	0.119
History			0.513
Endometrioid	19	13	
Nonendometrioid	2	0	
NA	3	0	
Grade			0.396
G1	2	5	
G2	6	4	
G3	7	4	
NA	9	0	
No. of bone lesions			0.985
Single	13	7	
Multiple	11	6	
Concomitant extraosseous metastases			0.708
Yes	8	3	
No	15	10	
Solitary bone metastasis without extraosseous involvement			0.501
Yes	8	6	
No	15	7	
Metastases to the axial skeleton			0.082
Yes	14	3	
No	10	10	
Overall survival, median, months	36.0	57.0	0.084

Abbreviations: NA, not available.

## Data Availability

Not applicable.
